# Editorial

**Published:** 1873-05

**Authors:** 


### Editorial.

### DIAGNOSIS.

The importance of correct diagnosis in cases of dental disease is often underrated ; and this is done not only by the student who is acquiring the elements of professional education, but by the regular practitioner, who is every day engaged in the treatment of cases.

Not only do physicians treat for the wrong disease, but dentists too make mistakes. A course of medication addressed to the heart, where the stomach alone is in fault, is no worse than an application of carbolic acid, to cure aching caused by a dead pulp in a sealed canal. Both are mistakes in practice, founded upon errors in diagnosis. That the practice of dentistry, as well as that of medicine, contains cases of faulty diagnosis is a matter of frequent observation.

And yet it is all important to the patient that a diagnosis of his case be made correctly, and at once. A mistake committed at this starting point must constitute the foundation for a long course of false medication. By diagnosis the character of the treatment is determined. If the diagnosis be incorrect, the treatment will not only not be salutary, but is almost certain to be pernicious. It is indeed better to do nothing than to do something which bears no curative relation to the morbid condition at which it is aimed. If the treatment be not curative in its influence, it is likely to be damaging. it thus appears that too much stress can not be put upon diagnosis. This is an advantage that must be secured to the patient, whether he be under the care of either the dentist or physician.

But if it be true that *correctness* of diagnosis is so *essential*, why are mistakes so often made ? It is because the making of a diagnosis is frequently the most difficult and perplexing thing which either the medical or dental practitioner is called upon to accomplish. There sometimes occur in the practice of the dental specialty cases of pain in the maxillary bones, periosteal disturbances in the roots of teeth, affection of the fifth pair of nerves, or discharges of pus from obscure sources, the correct understanding of which may well tax the highest powers of genius. When the morbid condition is once fixed upon and understood, the work is, for the most part, easy ; but to to tell *where* to treat and *what* to treat is the more difficult duty of the diagnostician.

In diagnosing an obscure case the practitioner must often summon to his aid all the anatomy, physiology, pathology, and histology, that he is master of. A patient comes, and is in pain. The most diligent inquiry after facts, the most rigid scrutiny of the sufferer's testimony, the keenest penetration, and the most rigid adherence to the ratiocinative and inductive methods of science, will not always conduct the inquirer to the true cause of the malady.

The numerous symptoms of the patient must not be considered in the order communicated ; they must be redistributed in the mind of the practitioner into their logical arrangement. The non-essential symptoms must be eliminated. Those which carry a real significance must be interpreted. Nor will it answer to give a too arbitrary interpretation, since the symptoms by which a disease is to be recognized are not invariable.

Many modifying circumstances and conditions are to be held constantly before the mind. Constitutional peculiarities and idiosyncrasies, temperament, diathesis, and heredity are a kind of personal mirror through which each patient must be viewed. These conditions, varying in different persons, proportionally modify the course of the *same* malady in *different* patients.

It is therefore true that those diagnosticians who have sought for pathognomonic symptoms have in general been in the wrong. There is no such thing as a pathognomonic symptom for every disease. Much depends on the mass of symptomatic evidence ; much on the conjunction of different symptoms ; much on the order of succession in which they arise. The absence of a symptom may be as significant as its presence.

But it is no part of my present intention to lay down the alws of diagnosis. I have only desired to record a single word in favor of a more careful study of the subject, and mastery of its methods. A suffering patient ,with a complicated assemblage of symptoms, presents a problem to the mind, which is not always an easy one. The path to its solution is often obscure, and difficult to trace ; from it lead off many by-paths that open broad enough, but conduct it to nothing; it is beset by many pitfalls of fallacy, and misleading delusion that make the diagnosis of a complicated case the crowning performance of the skilled dentist or physician.

### THE OHIO DENTAL COLLEGE.

Owing to unforseen and unavoidable circumstances there is no session held in this Institution this spring, as was intended at the close of last regular session. The vacation of two or three of the chairs occurring at that time it seemed to the remaining members of the faculty that an opportunity should be given the Board of Trustees to reorganize the Faculty entire, and make such changes as the best interests of the College might demand. So with this view the Faculty resigned as a whole. This was done with the most perfect harmony and good feeling by all parties ; and this was subsequently most fully evinced by the reappointment of all the members of the former Faculty whom circumstances would at all permit to accept.

In the accomplishment of this work so much time was lost as to render the holding of a spring session impracticable. This is a matter of regret to all the friends of the Institution ; our colleges should all be kept open for instruction during the greater part of the year.

The Chairs in the College are all filled by the former incumbents, except those of clinical and mechanical dentistry : appointments have been made for these, but we are not yet authorized to announce their acceptance. We are fully warranted in saying, however, that the Board will not rest satisfied with anything less than the occupancy of these chairs by the very best men.

The determination of all connected with the Institution is that its present standard shall be maintained ; and we are assured a higher degree of usefulness and efficiency will in the future be attained by virtue of enlarged experience, and the increased demand of the dental profession for an elevation of the standard of dental education.

The annual announcement of the Institution will be issued soon, to which we direct attention for details.

### LAWRENCE'S FILE HOLDER.

This is a very useful little instrument for the dentist, devised and constructed by Dr. H. Lawrence, of New Orleans. It is very simple in structure, and efficient in the performance of the work for which it is intended. It is a handle or holder, chiefly for thin separating files ; it holds equally well the entire file or any portion of it, even to half an inch in length, so that these files though broken in short pieces may be used, which was not the case with any holder previously in use. The file is held at an angle of from twenty-five to forty degrees, with the shaft of the instrument so that it will operate the file upon a point that a straight file or instrument will not touch. It also serves well as a holder for chisel or cutting instrument bits. The bite of the instrument is sufficiently strong to hold firmly any of these instruments. It should be in the case of every dentist. We presume it can be obtained at any of the dental depots.

### CALIFORNIA STATE DENTAL ASSOCIATION.

The California State Dental Association will commence its fourth annual session, in San Francisco, Tuesday May 20th, 1873, at 10 o'clock A. M., and continue four days. Every member of the profession on the Pacific Coast, is cordially invited to be present. Clinics daily.

H. J. PLOMTEAUX, Rec. Sec.

### HYMENIAL.

Married April 23, 1873, by Elder C. M. Blanchard, at the house of the bride's parents, Dr. E. M. Creditor of Angola, Ind., to Miss Emma Cox, of Kendallville, Ind.

May the largest amount of matrimonial bliss be theirs.

### ACKNOWLEDGEMENT.

We can not do less than make grateful acknowledgement for the very efficient and valuable aid rendered us in the editorial work in this and the two preceding numbers of the REGISTER, by very our worthy and scientific brother: Dr. D. C. Hawxhurst, recently of Battle Creek, Michigan, but now, and we hope henceforth of Cincinnati. If any body has wondered what has made the REGISTER start up with increased vigor recently, they have herein the explanation.—ED.

**LESSONS** IN PHYSICAL DIAGNOSIS, by Alfred M. Loomis, Professor of the Institutes and Practice of Medicine in the medical department of the University of New York; Physician to Bellevue and Charity Hospital, etc. Third Edition, revised and enlarged. W. WOOD & Co., New York, 1872.

This work is a series of lessons intended to put the student and young practitioner into possession of those physical methods of examination, which will be useful to him in detecting abnormal states of organs and tissues. It is concise and systematic, though drawn from a course of lectures given by the author to his medical classes in New York. It is probably the best work we have in its own department of physical examinations by palpitation, mensuration, succussions, percussion, ascultation, and by various instruments.

After treating of each of the six methods above mentioned, the author proceeds in successive chapters to give a synopsis of the physical signs of disease in the lungs, heart, abdomen, urinary organs, and nervous system.

Where special topics demand it, they have been treated with minuteness and fullness.

To the examination of urine, three Lessons are devoted, to chemical, volumetric and microscopic tests for abnormal elements, and remarks on their clinical significance. The author's description of chemical reagents, apparatus, and methods, is clear and intelligible.

The various instruments of physical diagnosis are described under the head of "Mechanical Aids to Diagnosis," occupying about thirty pages of the volume.

This work, though it can not take the place of the more philosophical works of DaCosta and others, will become a necessity to the student, on account of its thoroughness and excellence in its own department of physical examination, and is a valuable addition to our medical literature.

### DISCUSSION ON OFFENSIVE BREATH.

We presented our readers in the April number of the *Register*, with some discussions of the Ohio State Dental Association, on Fetid Breath. It is due to the various speakers as well as to Dr. Hawxhurst, to say that his paper, so frequently referred to, has not been published. The article on "Offensive Breath," printed in the March number of the *Register* is a very different production.

### MEETINGS OF THE CINCINNATI DENTAL ASSOCIATION.

The second and third meetings of this Association were held at Dr. Taft's office, on March 31st and April 15th, respectively, and were mainly devoted to a discussion of the Status of Dentistry in the City of Cincinnati. The subject was opened by L. P. Meredith. A most unusual interest was manifested in this question. Nearly all the members took part in the discussions; and the observation and experience of the Cincinnati profession were commented upon with much freedom.

D. C. Hawxhurst, Sec.

A joint meeting of the Illinois and Iowa State Dental Societies will be held at Rock Island and Davenport, on Tuesday, the 13th of May, and the three following days. Also a joint meeting of the Indiana and Kentucky State Societies in New Albany and Louisville, on the first Tuesday of June.

Let everybody in reach attend these meetings, as they will doubtless be grand occasions.

